# Molecular Weight Effect of Poly(dimethylsiloxane) on Its Interfaces with Water and Octane: A Molecular Dynamics Simulation Approach

**DOI:** 10.1021/acsomega.5c12788

**Published:** 2026-03-16

**Authors:** Arta Osmani, Mohammed Bazaid, Ji Yeon Kim, Eun Suk Shin, Seung Soon Jang

**Affiliations:** † School of Materials Science and Engineering, 1372 Georgia Institute of Technology, 771 Ferst Drive NW, Atlanta, Georgia 30332-0245, United States; ‡ Korea Dyeing and Finishing Technology Institute, 92, Dalseocheon-ro, Seo-gu, Daegu 41706, Republic of Korea

## Abstract

We investigate how the molecular weight of poly­(dimethylsiloxane) (PDMS) governs the interfacial behavior in contact with polar and nonpolar liquids using the molecular dynamics simulation method. PDMS chains with various degrees of polymerization (*n* = 9, 18, and 27) are simulated to quantify molecular-weight effects on density profile, interfacial tension, mobility, and segmental orientation. While the interfacial tensions for PDMS-water (40 dyn/cm) and PDMS-octane (10–12 dyn/cm) remain nearly independent of molecular weight, the microscopic structural responses exhibit clear chain-length dependence. Longer PDMS chains develop enhanced density fluctuations and layered packing near interfaces, reflecting reduced conformational freedom and stronger intrachain correlation. At PDMS-water interfaces, all systems form sharp boundaries, which is attributed to the hydrogen bonding network of the water phase, and PDMS adopts predominantly horizontal orientations to minimize unfavorable polar–nonpolar interactions. In contrast, PDMS-octane interfaces show broad, compositionally mixed regions where shorter PDMS chains more easily penetrate the hydrocarbon phase, consistent with the low interfacial tension and chemical compatibility. Mean-squared displacement analyses reveal a monotonic decrease in chain mobility with increasing molecular weight, with strong suppression near water and enhanced mixing at PDMS-octane interfaces. These results demonstrate that molecular weight crucially modulates interfacial structuring, conformational ordering, and chain dynamics, even when macroscopic thermodynamic properties remain unchanged. This molecular-level insight provides a predictive basis for engineering PDMS-based coatings, adhesives, and liquid-contacting surfaces with tunable interfacial performance across diverse chemical environments.

## Graphical abstract

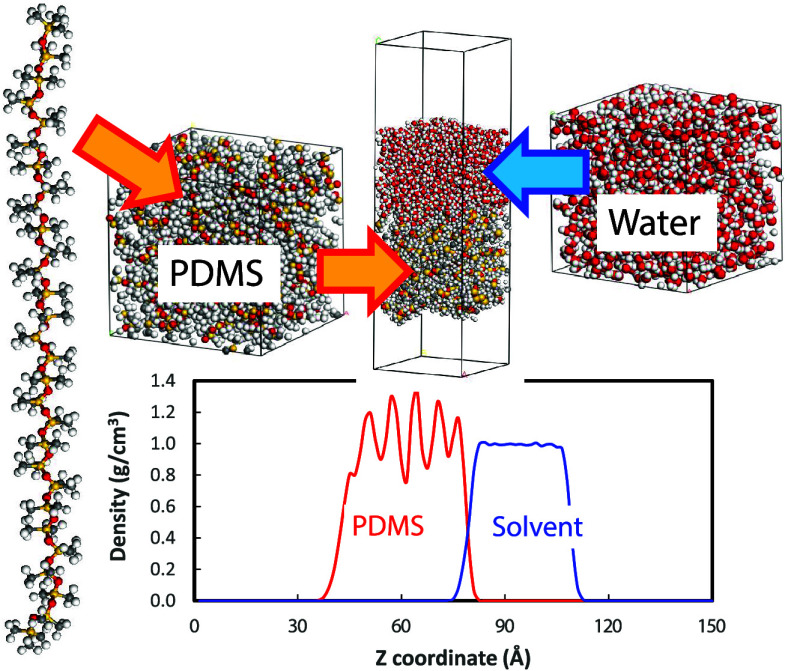

## 1 Introduction

Surface and interface science is central to the performance of a wide range of technologies, such as biotechnologies,^1^ energy technologies,^2^ coatings,^3^ adhesives,^4^ and sensors.^5^ The interfacial region represents a zone of chemical and physical heterogeneity, where molecular arrangement and intermolecular interactions dictate macroscopic properties, such as wettability,^6^ adhesion,^7^ and surface energy.^8^ Therefore, the design of advanced materials with tailored interfacial properties requires a comprehensive understanding of the molecular-level phenomena occurring at these boundaries. Recent advances in both experimental characterization^9,10^ and computational modeling^11^ have enabled probing these complex interfacial systems with unprecedented resolution.^12^

For decades, fluorocarbon-based surface treatments have been the benchmark for achieving low surface energy^13,14^ and superhydrophobicity.^15^ Their unique performance stems from the strong electronegativity of fluorine atoms and the extended perfluorinated chains that minimize intermolecular interactions.^16^ However, mounting environmental and health concerns associated with the persistence and bioaccumulation of perfluoroalkyl substances have prompted urgent efforts to identify safer alternatives.^17^

In this context, poly­(dimethylsiloxane) (PDMS) has emerged as an attractive candidate. PDMS combines a flexible Si–O backbone with methyl side groups that confer low intermolecular cohesion, resulting in low surface tension and desirable interfacial properties. Moreover, PDMS offers low glass transition temperature, high chemical and thermal stability, and strong adhesion to diverse substrates,^1,18,19^ enabling its broad adoption in adhesives,^20^ protective coatings,^21^ and encapsulants.^22^

The interfacial behavior of PDMS is not determined solely by its chemical composition but also by its molecular architecture and chain conformations.^18^ In polymer systems, molecular weight strongly influences physical properties such as chain entanglement, mobility, and surface segregation.^23^ For example, studies on other polymer-liquid interfaces have shown that increasing chain length can alter interfacial tension, change density profiles, and influence wettability by modifying how chains pack and orient at the interface.^24^ For PDMS, however, the effect of molecular weight on interfacial interactions with polar and nonpolar liquids has not been thoroughly characterized. Given the extensive use of PDMS in liquid-contacting applications, this lack of molecular-level insight represents a significant gap in the field.

Experimental studies of PDMS interfaces have primarily relied on contact angle measurements^25^ and pendant drop techniques^26^ to quantify surface or interfacial tension. While these macroscopic methods provide important benchmarks, they cannot fully elucidate the molecular-scale mechanisms governing interfacial stability. For instance, the adsorption of small molecules at PDMS surfaces, the time-dependent evolution of interfacial tension, and swelling phenomena in PDMS-alkane mixtures have been reported.^27,28^ However, the microscopic origins of these behaviors remain unclear. Furthermore, while swelling and solubility of PDMS in lower alkanes (C6–C12) have been studied experimentally, direct characterization of PDMS–octane interfaces across different molecular weights has yet to be achieved. Thus, the molecular-level determinants of PDMS interactions with both high-surface-tension liquids, such as water, and low-surface-tension liquids, such as octane, remain unclear.

Molecular dynamics (MD) simulations provide a robust framework to address these challenges. Unlike macroscopic measurements, MD simulations capture atomistic detail of chain conformations, intermolecular interactions, and time-dependent fluctuations at interfaces.^14,29–31^ They enable direct calculation of density profiles, orientation distributions, and interfacial tension, while also allowing systematic variation of molecular weight, temperature, or interfacial composition. Previous MD studies have offered valuable insights into related systems.^32^ For example, simulations of PPS surfaces with zinc oxide nanoparticles demonstrated the interplay of hydrogen bonding and van der Waals forces in determining hydrophilic versus hydrophobic character.^33^ Investigations of water-octane interfaces revealed how molecular orientation and coordination numbers govern interfacial thickness and inhomogeneity.^34^ In PDMS systems, simulations have been used to study crystallization, chain relaxation, grafting behavior, and water wettability.^18,35,36^ Coarse-grained models have approximated contact angles in agreement with experiment, while atomistic approaches have more directly resolved interfacial tension. However, the role of PDMS molecular weight in shaping interfacial properties with both water and alkanes remains relatively unexplored.

In this study, we employ all-atom MD simulations to systematically investigate the interfaces of PDMS with water and octane. By simulating PDMS chains of different molecular weights at equilibrated interfaces, we evaluate interfacial tension, density distributions, chain conformations, scattering profiles, and hierarchical chain ordering. Our investigation centers on the oligomeric regime (*n* = 9–27) to isolate the fundamental thermodynamic drivers of interfacial structuring without the confounding factors of topological entanglement. This range allows us to explicitly observe critical features, such as the saturation of density fluctuations and the role of mobile chain ends, which are often obscured in high-molecular-weight chains. Consequently, the trends established here offer valuable insight into the local physical interactions that govern adhesion and wettability, guiding how longer chains will pack and orient at the immediate interface. Through these simulations, we aim to clarify how molecular weight dictates PDMS behavior in contact with polar and nonpolar liquids, improving our understanding toward the rational design of PDMS-based materials for coatings, adhesives, and repellent surfaces. By situating PDMS within the larger context of surface science and highlighting its unique advantages over traditional fluorocarbons, this work advances both the methodological framework and the fundamental understanding of polymer-liquid interfaces.

## 2 Models and Simulation Methods

### 2.1 Force Fields and Simulation Conditions

In this study, molecular interactions were described using the DREIDING force field^37^ for PDMS and octane, and the F3C model^38^ for water. The DREIDING force field was selected for its balance of computational efficiency and accuracy in modeling organosilicon polymers. While Class II force fields such as COMPASS offer higher-order cross-terms, DREIDING has been successfully employed in numerous studies^29,31^ to reproduce the structural and thermodynamic properties of soft matter systems. Furthermore, recent benchmarking studies have demonstrated that general-purpose force fields like DREIDING provide reliable predictions for PDMS density and interfacial tension that are consistent with experimental data, particularly for the nonbonded interactions that dominate interfacial behavior.^39^ The DREIDING force field has the following total potential energy form:

1 $$E_{total}=E_{vdW}+E_{Q}+E_{bond}+E_{angle}+E_{torsion}+E_{inversion}$$

where *E*_total_, *E*_vdW_, *E*_Q_, *E*_bond_, *E*_angle_, *E*_torsion_, and *E*_inversion_ denote energies for total, van der Waals, electrostatic, bond stretching, angle bending, torsion, and inversion components, respectively. The atomic charges for PDMS and octane molecules were assigned via Mulliken population analysis^40^ at the B3LYP/6-31G** level using Jaguar,^41^ while water charges were obtained directly from the F3C model.^38^ The equations of motion were integrated using the velocity Verlet algorithm^42^ with a time step of 1 fs. Temperature and pressure were regulated using the Nose-Hoover thermostat and barostat.^43–45^ We employed the large-scale atomic/molecular massively parallel simulator (LAMMPS) code^46^ for all simulations.

### 2.2 Model Construction

Figure 1 shows the chemical structure of PDMS with three different molecular weights. Using PDMS chains in Figure 1b–d, we built amorphous bulk phase PDMS systems as shown in Figure 2a and equilibrated the systems. Then we built slab systems by extending the *z*-axis dimension as shown in Figure 2b. The degree of polymerization, number of chains, and dimensions of each system are summarized in Table 1. Similarly, we built and equilibrated bulk phases of water and octane, then built slab structures, as shown in Figure 2b,e and c,f, respectively. Next, to investigate the interfaces of PDMS phase with water and octane phases, we constructed binary slab systems as shown in Figure 3a,b, respectively, and water-octane system (Figure 3c) as a reference for further analysis.

**Figure 1:**
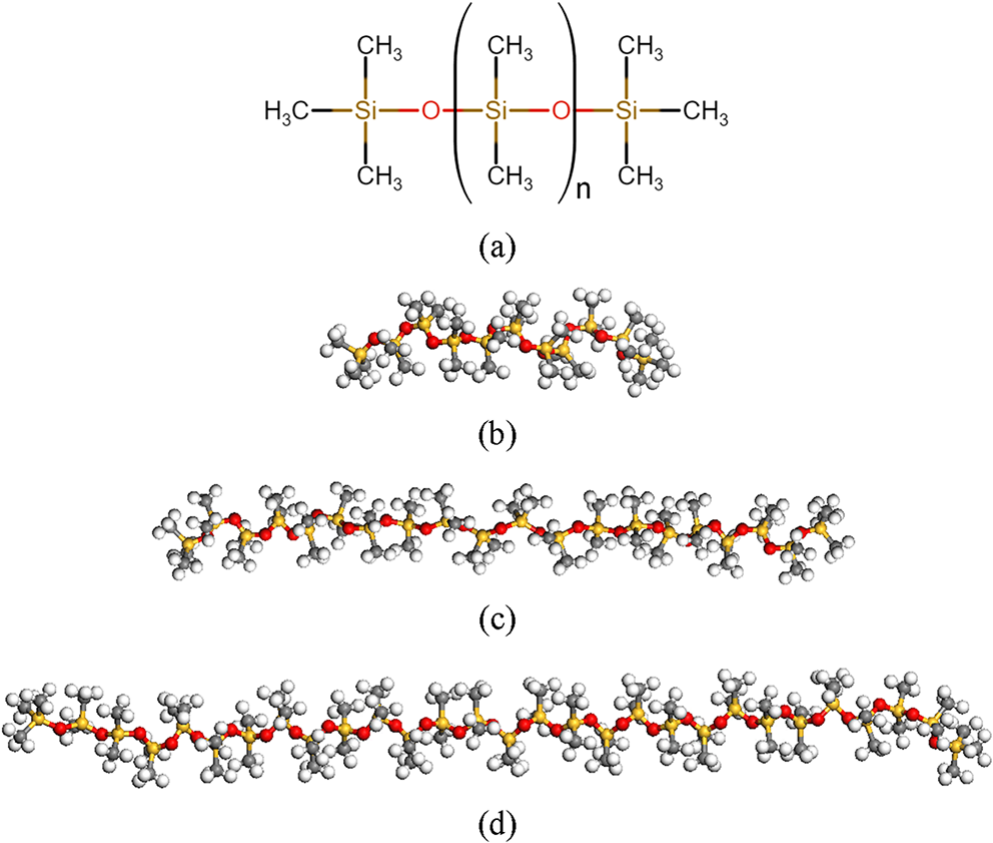
(a) Chemical structure of PDMS and PDMS chain models with (b) *n* = 9, (c) *n* = 18, and (d) *n* = 27 repeat units. Orange, red, gray, and white colors denote silicon, oxygen, carbon, and hydrogen atoms, respectively.

**Figure 2:**
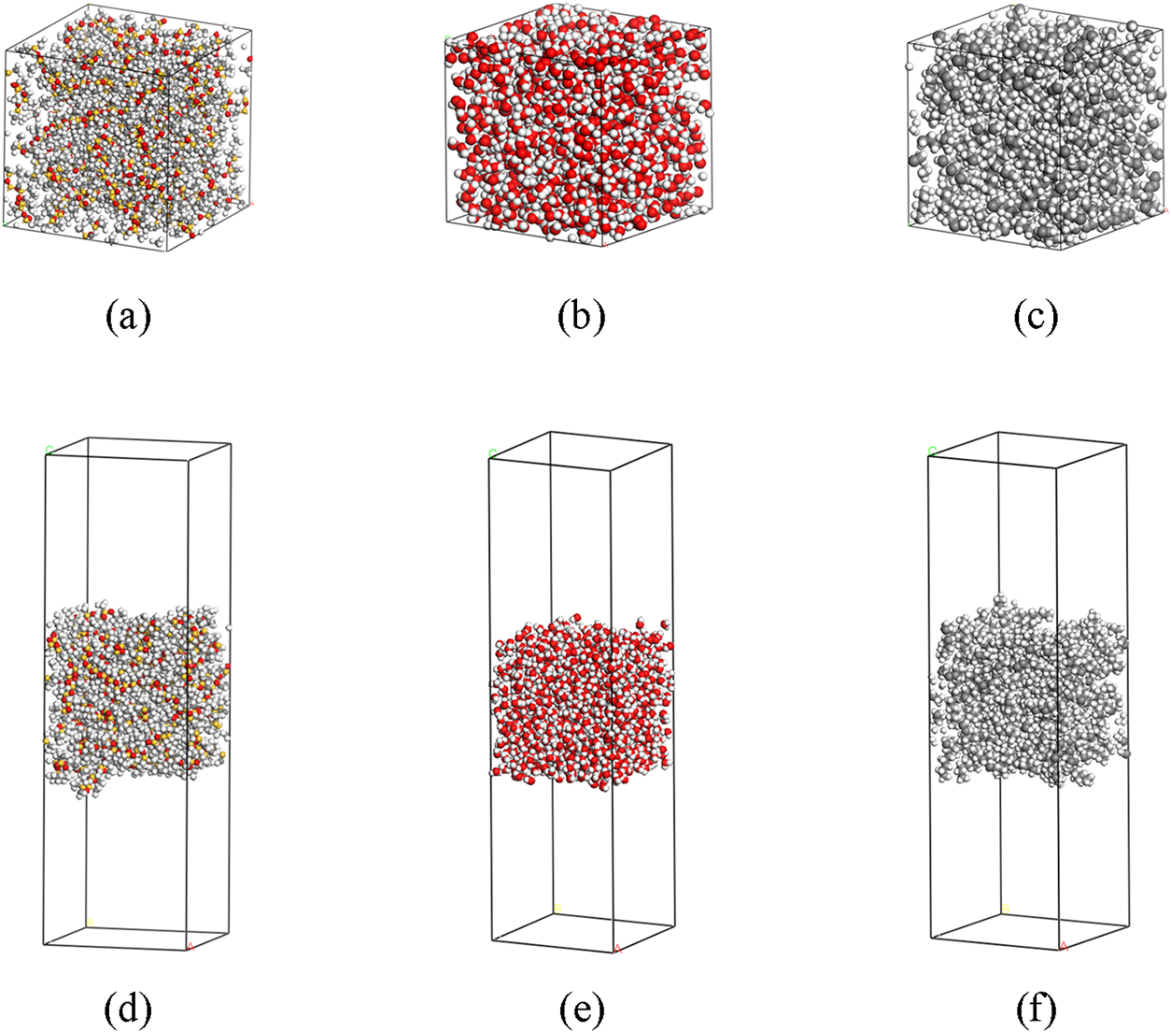
(a) PDMS bulk phase; (b) water bulk phase; (c) octane bulk phase; (d) PDMS slab; (e) water slab; (f) octane slab.

**Figure 3:**
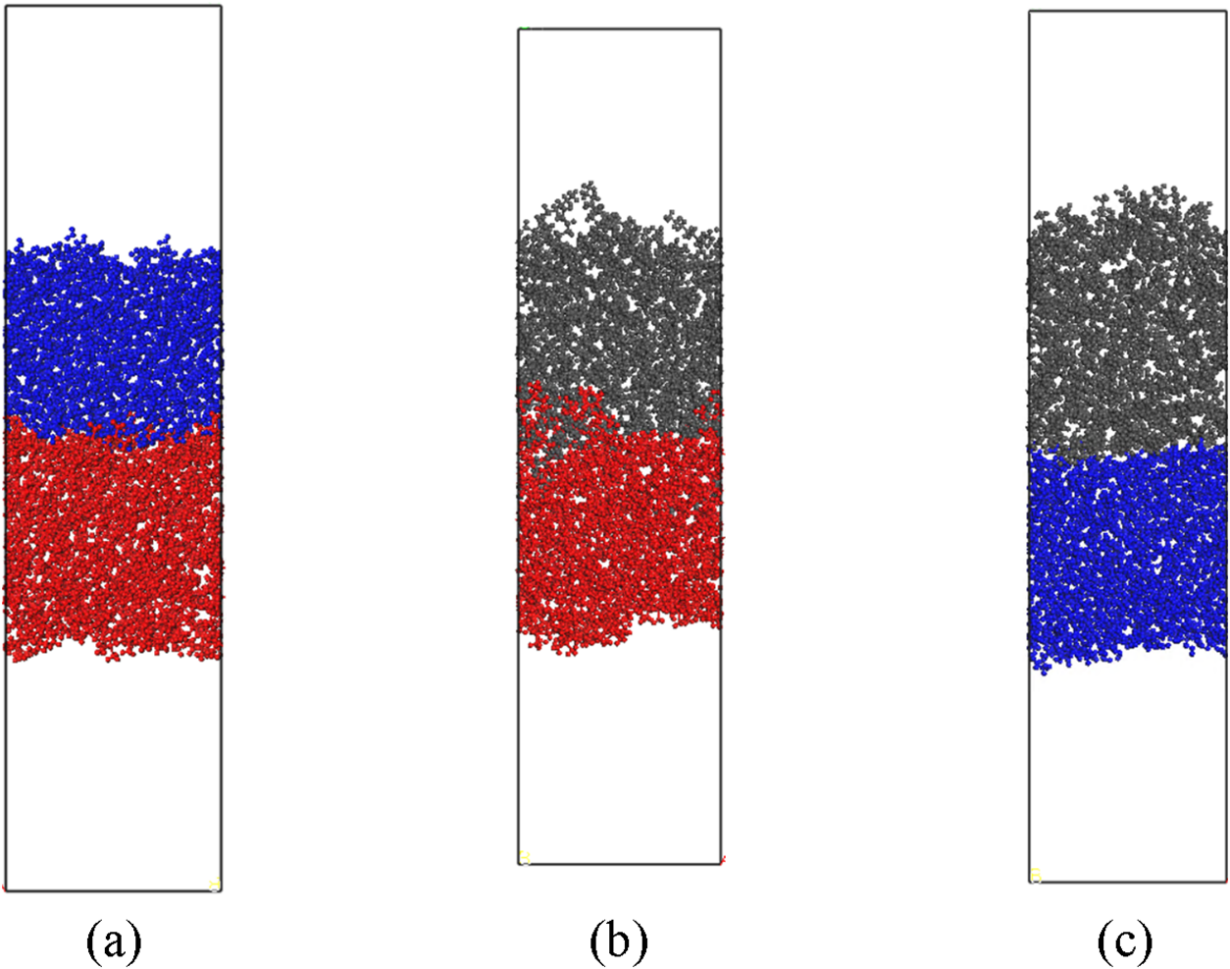
Interfacial systems: (a) PDMS-water; (b) PDMS-octane; (c) water-octane. The red, blue, and gray colors indicate PDMS, water, and octane, respectively. PDMS18 is used to represent PDMS. PDMS9 and PDMS27 show similar features.

**Table 1:** Summary of Simulated Systems

**systems**	**degree of polymerization**	**number of molecules**	**system dimensions**
**PDMS9**	9	35	bulk: 36.2 × 36.2 × 36.2 Å^3^
			slab: 36.2 × 36.2 × 120 Å^3^
**PDMS18**	18	20	bulk: 36.4 × 36.4 × 36.4 Å^3^
			slab: 36.4 × 36.4 × 120 Å^3^
**PDMS27**	27	14	bulk: 36.2 × 36.2 × 36.2 Å^3^
			slab: 36.2 × 36.2 × 120 Å^3^
**water**		1301	bulk: 34.0 × 34.0 × 34.0 Å^3^
			slab: 34.0 × 34.0 × 120 Å^3^
**octane**		147	bulk: 35.7 × 35.7 × 35.7 Å^3^
			slab: 35.7 × 35.7 × 120 Å^3^

The PDMS bulk-phase simulations were performed in three stages: initial thermalization, annealing, and equilibration. In the initial thermalization stage, each bulk phase system was first heated from 10 to 298 K using velocity rescaling and stabilized using NVT MD simulation method for 1 ns and subsequently NPT MD simulation for 2 ns. For the annealing stage, the following three steps were performed in a cycle: first, the system was heated to 600 K with a volume expansion to 200% of the target volume; second, the NVT MD simulation was implemented at 600 K; third, the system was cooled to 298 K with a volume compression to 100% of the target volume. Then, this cycle was repeated three times to obtain a more relaxed structure for each system. After the annealing stage, the system was compressed to 80% of the target volume. During the annealing stage, we used a direct cutoff method to compute Coulombic interactions. After the annealing stage, we performed NVT MD simulation for 1 ns and subsequently NPT MD simulation for 260 ns to complete the equilibration stage. During this extended equilibrium stage, we employed the particle–particle-particle-mesh (PPPM) method for the long-range correction of Coulombic interactions and used the PPPM method for all subsequent computations.

Once we completed the equilibration of the PDMS bulk phase system, we extended the *z*-axis dimension to 120 Å, thereby generating a slab configuration with empty space. Since the initial slab structure has unequilibrated surface structures, we performed an NVT MD simulation for 50 ns to further equilibrate the slab system. Please note that the slab was located at the center of the simulation box.

Next, we combined two slab systems in a simulation box to generate PDMS-water, PDMS-octane, and water-octane interfaces. After equilibration, we performed MD simulations for 30 ns and analyzed the systems using the last 10 ns segment in the equilibrium state. For all NVT slab simulations, for both pure and interfacial systems, we utilized parameters of 15 Å for the Lennard-Jones cutoff and 18 Å for long-range electrostatic interactions.

### 2.3 Calculation of Interfacial Tension

To determine the interfacial properties of various systems, we calculate the interfacial tension from MD simulations of single-component slabs (PDMS, water, and octane in vacuum) and interfacial systems (PDMS-water, PDMS-octane, and water-octane). The calculation is based on the definition of interfacial tension, which arises from the anisotropy of the pressure tensor at an interface. From a thermodynamic perspective, the interfacial tension (γ) is defined as the change in Gibbs free energy (*G*) with respect to an infinitesimal, reversible change in the interfacial area (*A*) at constant temperature (*T*), pressure (*P*), and composition (*n*):

2 $$\gamma ={\left(\frac{\partial G}{\partial A}\right)}_{T,P,n}$$

defining γ as the free energy change per the surface area change.

In a bulk, isotropic fluid at equilibrium, the system is uniform in all directions. Consequently, the diagonal components of the pressure tensor are equal on average:

3 $$\left\langle P_{\mathrm{xx}}\right\rangle =\left\langle P_{\mathrm{yy}}\right\rangle =\left\langle P_{\mathrm{zz}}\right\rangle$$

However, the presence of an interface breaks this symmetry. For an interface lying in the *xy*-plane, the molecular environment is no longer uniform along the *z*-axis. This inhomogeneity creates a pressure anisotropy, where the pressure component normal to the interface (*P*_*zz*_) is different from the components tangential to it (*P*_*xx*_ and *P*_*yy*_).

The relationship between this pressure anisotropy and surface tension can be shown by considering the work done to change the interfacial area. The surface tension γ is the integral of the difference between the normal and tangential pressure profiles across the interfacial region. For a simulation box of length *L*_*z*_, this relationship is expressed in the practical formula used for computation, which connects the macroscopic property of interfacial tension to atomistic-level pressure fluctuations.

For a simulation of a single material slab surrounded by a vacuum (e.g., PDMS, water, or octane), there are two identical material-vacuum interfaces. The total interfacial tension for the entire simulation box is calculated using the Kirkwood-Buff theory:^47^

4 $$\gamma_{total}=L_{z}(\left\langle P_{\mathrm{zz}}\right\rangle -\frac{\left\langle P_{\mathrm{xx}}\right\rangle +\left\langle P_{\mathrm{yy}}\right\rangle }{2})$$

where γ_total_ is the combined interfacial tension of both interfaces in the simulation box, *L*_*z*_ is the length of the simulation box along the *z*-axis (perpendicular to the interface), *P*_*xx*_, *P*_*yy*_, and *P*_*zz*_ are the diagonal components of the pressure tensor. The formula denotes a time average taken over an equilibrated segment from the end of the simulation trajectory.

Since the simulation box contains two identical interfaces, the interfacial tension for a single material-vacuum interface, γ_M‑vac_, is simply half of the total tension:

5 $$\gamma_{M‐\mathrm{vac}}=\frac{1}{2}\gamma_{\mathrm{total}}$$

where *M* denotes material such as PDMS, water, and octane.

To find the interfacial tension between two different materials, such as PDMS and water (γ_PDMS‑water_), we used binary slab systems. The total interfacial tension of a system is the sum of the interfacial tensions. For a PDMS-water system with vacuum on either side, the total interfacial tension includes contributions from the PDMS-vacuum interface, the water-vacuum interface, and the PDMS-water interface.

6 $$\gamma_{\mathrm{total}\left(s\mathrm{ystem}\right)}=\gamma_{\mathrm{PDMS}‐\mathrm{vac}}+\gamma_{\mathrm{PDMS}‐\mathrm{water}}+\gamma_{\mathrm{water}‐\mathrm{vac}}$$

To isolate the value of γ_PDMS‑water_, we can rearrange this equation. This requires running three separate simulations: a simulation of the combined PDMS-water system to calculate γ_total(system)_, a simulation of a PDMS slab with vacuum to calculate γ_PDMS‑vac_, a simulation of a water slab with vacuum to calculate γ_water‑vac_. With the results from all three simulations, the interfacial tension between PDMS and water can be calculated as

7 $$\gamma_{\mathrm{PDMS}‐\mathrm{water}}=\gamma_{\mathrm{total}\left(\mathrm{system}\right)}-{\gamma_{\mathrm{PDMS}‐\mathrm{vac}}+\gamma }_{\mathrm{water}‐\mathrm{vac}}$$

For each calculated interfacial tension value, the pressure tensor data were obtained from an MD simulation run in the NVT (canonical) ensemble. The pressure data were averaged over the final 10 ns segment of the simulation trajectory to ensure the analysis was performed on a fully equilibrated system where thermodynamic properties were stable. For clarity regarding unit conventions, we note that 1 dyn/cm is equivalent to 1 mN/m. Throughout this study, we consistently report interfacial tension values in dyn/cm. To ensure statistical reliability, the reported interfacial tension and density values were obtained by averaging results from multiple independent simulations. The associated uncertainties correspond to the standard deviation across these trials.

### 2.4 Calculation of Orientation Function

We analyze the segment orientation of PDMS chains using the Orientation Function (S). This function is derived from the second-order Legendre polynomial, P_2_, and provides a quantitative measure of molecular orientation. The S is calculated using the following equation:

8 $$S=\left\langle P_{2}\left(\cos\delta \right)\right\rangle =\frac{1}{2}\left(3\left\langle \cos^{2}\left(\delta \right)\right\rangle -1\right)$$

where δ is the angle between a vector representing a segment of the polymer chain and a reference axis. For interfacial systems, this reference is typically the *z*-axis, which is perpendicular to the interface plane (the *xy*-plane). The angle brackets denote a time average taken over an equilibrated segment in the simulation.

The value of *S* provides a clear interpretation of the average chain alignment. *S* = 1 indicates a perfect alignment parallel to the *z*-axis (δ = 0), meaning that the polymer chains are oriented perpendicular to the interface, often described as “standing up”. *S* = −0.5 indicates a perfect alignment perpendicular to the *z*-axis (δ = 90), meaning that the chains are lying flat, parallel to the *xy*-plane of the interface. *S* = 0 indicates a completely random orientation, with no preferred direction of alignment, which is characteristic of a or isotropic bulk phase. To apply this analysis, the orientation of the PDMS chains must be quantified. This is achieved using segment vectors, allowing for the calculation of the segment orientation relative to the *z*-axis. For this purpose, we define the segment vector as a vector connecting the *i*-th Si to the (*i*+4)-th Si atom along the PDMS backbone, comprising five successive Si atoms for each *i* along the backbone: we refer it as the 5-Si segment vector.

The definition of the segment vector was carefully determined to balance the resolution of conformational details with statistical robustness. Preliminary analyses using shorter vector lengths (e.g., 2-Si or 3-Si) were found to be dominated by local bond vibrations and angle fluctuations, resulting in noisy orientation profiles that obscured the effective trajectory of the polymer backbone. Conversely, defining vectors over significantly longer intervals showed too coarse an approximation, effectively flattening the chain and failing to capture local conformational features, such as loops or sharp folds in the chains. The chosen 5-Si segment vector represents an optimal intermediate scale: it filters out local bond noise while retaining the sensitivity needed to resolve the chain conformational response to the interface. Even for the shortest oligomers (*n* = 9), this vector length allows for multiple overlapping segments per chain, ensuring sufficient data points for adequate statistical sampling. For the longer chains (*n* = 18, 27), the 5-Si vector provides a robust description of chain conformation, accurately reflecting the segmental alignment without oversimplifying the paths of polymer chains.

By calculating the cosine of the angle for each of these segment vectors (5-Si vectors) with respect to the *z*-axis, we generate a segment orientation profile. This profile provides statistical information on segment orientation as a function of segment location along the *z*-axis in the simulated systems. This analysis allows for a direct, quantitative comparison of how the segments in the polymer chain are geometrically arranged in the interfacial region. Furthermore, by analyzing these profiles at different time stamps from the MD simulation, we study the time-dependent behavior of chain conformations. This approach is critical for understanding how structural properties, such as chain alignment at an interface, are influenced by factors such as molecular weight.

## 3 Results and Discussion

### 3.1 Density Profiles and Interfacial Tensions

First, we prepared slab systems of PDMS, water, and octane via NVT MD simulations, as shown in Figure 2d,e,f, respectively, and then combined two different slabs in a system and ran NVT MD simulations to obtain PDMS-water, PDMS-octane, and water-octane interface systems as shown in Figure 3a,b,c, respectively. The interfacial thicknesses are calculated using the “80–20” rule at vacuum interfaces and “80–80” rule between two phases,^29,31^ and are summarized in Table 2.

**Table 2:** Simulated Interfacial Thickness Values

**solvents**	**systems**	**interfacial thickness (Å)**
**vacuum**	**water**	2.6 ± 0.9
	**octane**	5.4 ± 1.9
	**PDMS9**	3.2 ± 1.2
	**PDMS18**	7.4 ± 0.4
	**PDMS27**	6.2 ± 0.9
**water**	**PDMS9**	3.2 ± 0.7
	**PDMS18**	3.0 ± 0.7
	**PDMS27**	2.8 ± 0.5
**octane**	**PDMS9**	8.0 ± 2.3
	**PDMS18**	6.6 ± 4.0
	**PDMS27**	6.1 ± 3.4
**water-octane**		3.1 ± 0.8

#### 3.1.1 PDMS-Vacuum Interface

From Figure 4a, we found that the fluctuation in the PDMS density profiles increases with increasing molecular weight although the average densities in the slab are similar regardless of molecular weight: 1.02 ± 0.08 g/cm^3^ for PDMS9, 1.04 ± 0.12 g/cm^3^ for PDMS18, and 1.06 ± 0.17 g/cm^3^ for PDMS27. Across the pure PDMS systems, the interfacial thickness of PDMS18 and PDMS27 are larger than that of PDMS9 (PDMS9:3.2 ± 1.2 Å; PDMS18:7.4 ± 0.4 Å; PDMS27:6.2 ± 0.9 Å), attributed to the molecular-weight-dependent chain packing at the interface.

**Figure 4:**
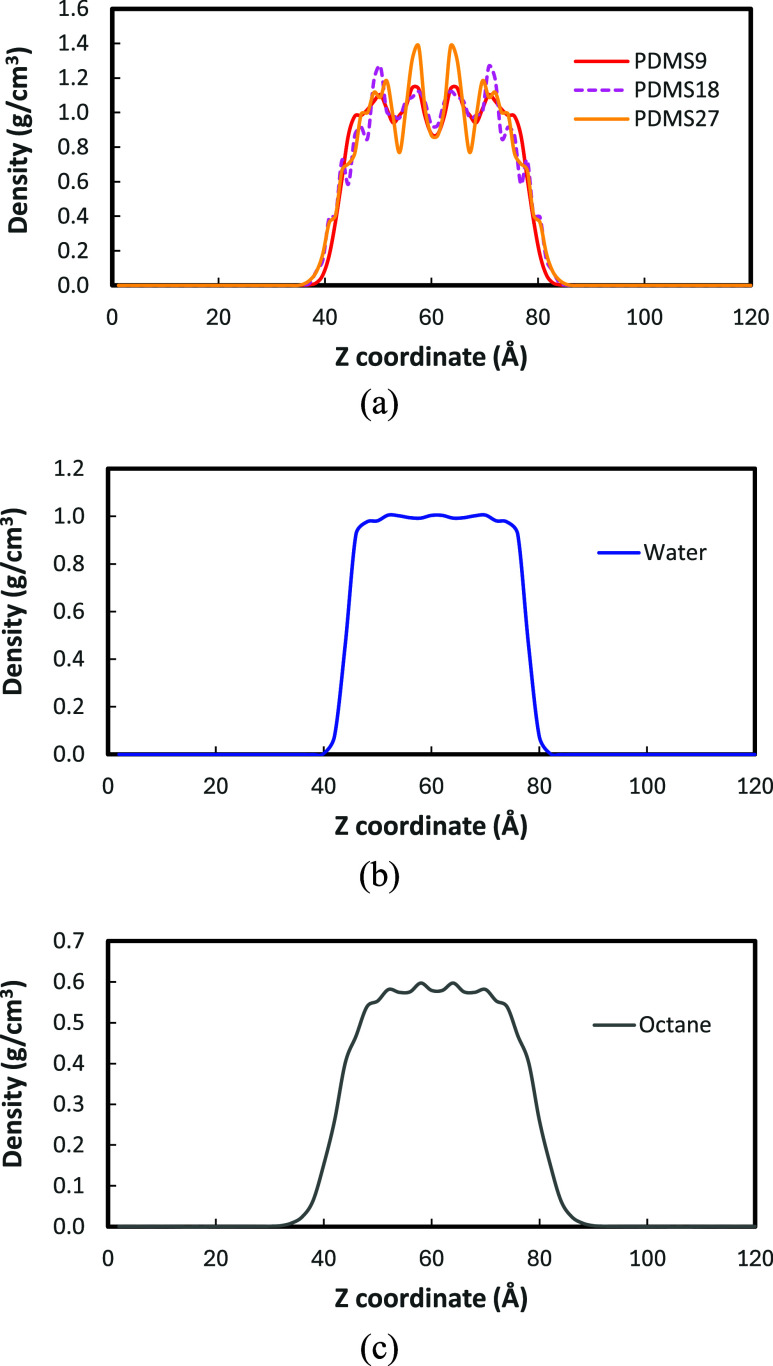
Density profiles for slab systems: (a) PDMS (*n* = 9, 18, 27) systems; (b) water system; (c) octane system.

In Figure 4a, we found that the PDMS9 system exhibits the least pronounced fluctuations, indicating a relatively even distribution of chain segments throughout the slab. In contrast, PDMS18 displays more distinct layering, and PDMS27 shows the most significant fluctuation, suggesting that the slab system exhibits packing and ordering of chains with increasing molecular weight. This observation in the density profiles reflects the balance between molecular mobility and chain connectivity. The shorter PDMS9 chains have greater conformational diversity and higher mobility, enabling them to adopt a broader range of orientations and maintain a more uniform density distribution. As chain length increases, the greater covalent connectivity and entanglement restrict segmental motion, leading to enhanced local ordering and reduced configurational entropy. The resulting structure**s** in PDMS18 and especially PDMS27 indicate the emergence of layered organization within the pure phase, consistent with a more ordered polymer packing.

From a thermodynamic perspective, shorter PDMS chains gain an entropic advantage due to their configurational diversity, leading to a less structured density profile. Conversely, longer chains incur an entropic penalty when attempting to reorient, favoring more regular and spatially correlated arrangements. These findings suggest that chain length not only influences the mechanical flexibility and relaxation dynamics of PDMS but also governs the intrinsic degree of molecular ordering even within its pure phase. It is important to note that these distinct oscillatory density fluctuations are likely characteristic of the oligomeric regime, where finite-size effects magnify the local packing order. While the fundamental segmental motifs established here serve as a baseline for PDMS interfacial behavior, we anticipate that in the high-molecular-weight limit, the macroscopic density profile would become smoother. In such entangled systems, topological constraints and statistical averaging over larger volumes would dampen these density oscillations, even though the local interfacial density remains consistent.

Regarding interfacial tension, as summarized in Table 3, we find that our simulated values for the PDMS systems are consistent across all molecular weights, averaging 23 dyn/cm, in good agreement with values reported in both experimental studies^48^ (21 dyn/cm) and previous simulations^18^ (20.5–26 dyn/cm). This consistency further confirms that molecular weight has no significant effect on PDMS surface tension within the range studied.

**Table 3:** Simulated Interfacial Tension Values for Pure Systems and Binary Systems

**system name**	**γ** (dyn/cm)	**γ**_**PDMS‑water**_ (dyn/cm)	**γ**_**PDMS‑octane**_ (dyn/cm)
**PDMS9**	22.9 ± 2.79	39.4 ± 3.9	12.1 ± 4.8
**PDMS18**	22.7 ± 2.81	40.0 ± 3.2	10.6 ± 4.5
**PDMS27**	22.8 ± 3.01	40.2 ± 3.4	12.4 ± 4.9
**water**	67.1 ± 1.64	**γ**_**water‑octane**_ = 49.5 ± 2.48	
**octane**	19.8 ± 1.79		

#### 3.1.2 Water-Vacuum and Octane-Vacuum Interfaces

The density profiles of pure water and octane in Figure 4 exhibit well-defined bulk regions with characteristic liquid-vacuum interfaces. Water exhibits a stable bulk density of 0.99 ± 0.01 g/cm^**3**^ and forms a relatively sharp interface with an interfacial thickness of 2.6 ± 0.9 Å, consistent with its strong cohesiveness due to its hydrogen-bonding network. Octane shows a lower bulk density of 0.57 ± 0.02 g/cm^3^ and a broader interface of 5.4 ± 1.9 Å, reflecting weaker intermolecular interactions and looser molecular packing. Small fluctuations in the octane profile arise from short-range ordering. Overall, the agreement with expected bulk densities confirms the reliability of the simulation setup and provides a solid baseline for comparing PDMS-liquid interfaces.

The similar interfacial thickness measured for water-octane interface (3.1 ± 0.3 Å) indicate that this sharp interface results from phase separation between the water and octane phases. Because water has strong cohesive interactions and a high surface tension, it forms a relatively compact interface with nonpolar molecules such as octane.

For the water system, our calculated value of 67.1 dyn/cm aligns well with the literature, which reports a wide range of values^29,31,49–53^ from simulations (61.8–70.9 dyn/cm) and experiments (70.9–72.0 dyn/cm). This result falls squarely within the established range and is notably closer to the experimental measurements, indicating that the employed simulation model captures the interfacial properties of water with high fidelity. For octane, we obtained a surface tension of 15.4 dyn/cm (Figure 2f), which is in reasonable agreement with experimental results^54,55^ (18.4–23.4 dyn/cm) and prior simulations^56^ (20.4 dyn/cm), further supporting the reliability of our simulation protocol.

#### 3.1.3 PDMS-Water Interfaces

From the equilibrated PDMS-water interfaces (Figure 3a), we obtained the density profiles as shown in Figure 5. We found that PDMS-water interfaces commonly exhibit a well-defined interface, regardless of molecular weight variation. By analyzing the density profiles, we characterized the interfacial thicknesses (Table 2), which showed similar values across PDMS molecular weight: 3.2, 3.0, and 2.8 for PDMS9-water, PDMS18-water, and PDMS27-water, respectively. Further, we analyzed the individual interfacial thicknesses of the PDMS-water interface, as summarized in Table 2. The overall PDMS-water interfacial thickness remains comparable to the corresponding water-vacuum values, indicating that water largely dictates the sharpness of the boundary. The water molecules preserve their robust hydrogen-bonding network, with strong cohesive interactions that inhibit mixing with the PDMS phase. This effect, coupled with the hydrophobic nature of the PDMS methyl groups, constrains the polymer to a narrow interfacial region. In contrast, the PDMS-vacuum interfaces systematically broaden with molecular weight (from 2.8 to 5.2 Å), likely due to enhanced configurational freedom of the chain ends and reduced packing constraints at the vacuum interface.

**Figure 5:**
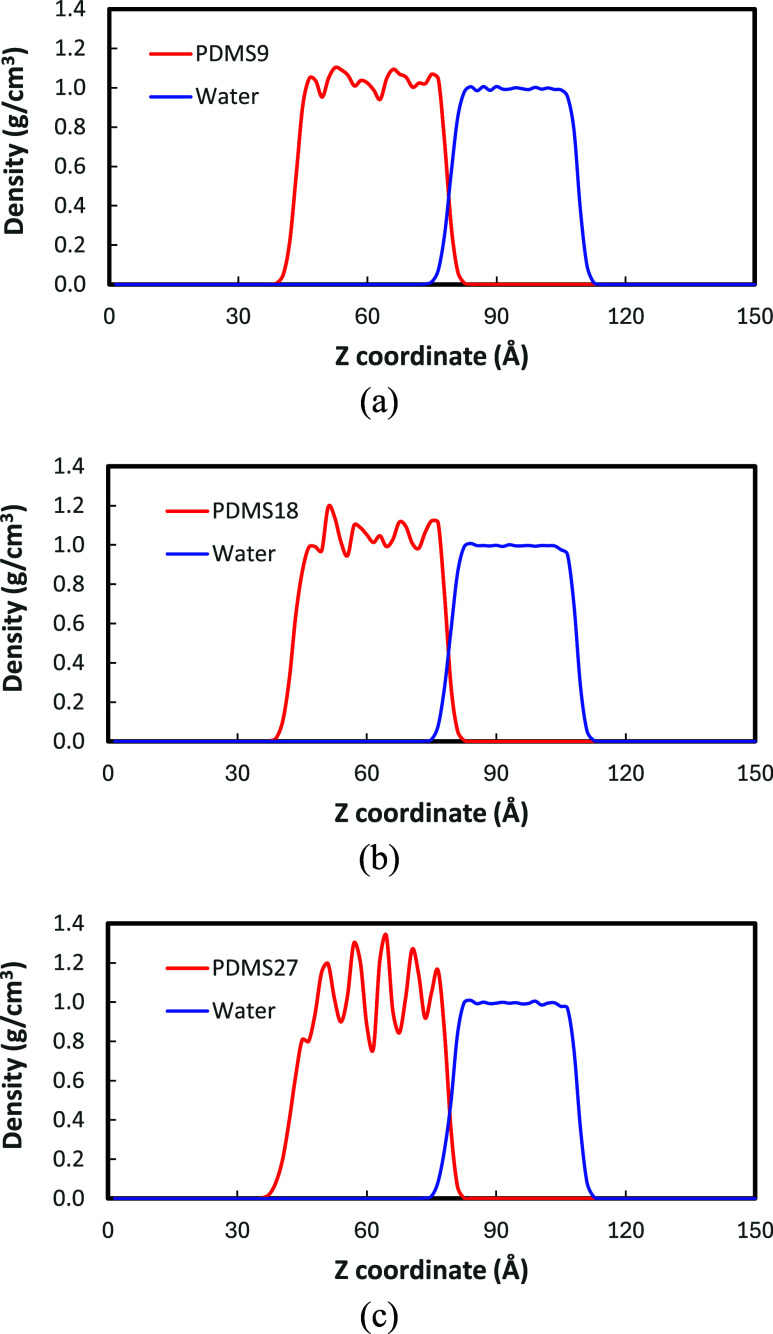
Density profiles for PDMS-water interfaces: (a) PDMS9; (b) PDMS18; (c) PDMS27.

Another noteworthy feature in Figure 5 is that the density fluctuates with increasing chain length. Such fluctuations correspond to layering structures within the PDMS phase, reflecting increased structural ordering. For the most extended chains (PDMS27), the periodicity in the density fluctuations becomes even more pronounced, suggesting a higher degree of structural organization and potentially reduced molecular mobility. To quantify this structural ordering, we calculated the maximum peak-to-valley amplitude of the density fluctuations within the slab. The amplitude increases systematically with chain length: approximately 0.12 g/cm^3^ for PDMS9, 0.25 g/cm^3^ for PDMS18, and 0.55 g/cm^3^ for PDMS27. This increase in density variation confirms that higher molecular weight chains pack more efficiently into distinct layers, whereas the shorter PDMS9 chains exhibit a more fluid-like, homogeneous bulk density. Again, this trend is consistent with the features observed in the PDMS-vacuum systems, where increasing molecular weight leads to more distinct fluctuations in the density profile. The vacuum interfaces exhibited a similar progression in density layering, with peak-to-valley amplitudes rising from approximately 0.15 g/cm^3^ for PDMS9 to 0.60 g/cm^3^ for PDMS27, as shown in Figure 4a. The similarity in the density profiles between the PDMS-vacuum and PDMS-water interfacial systems reinforces that the observed structuring is an intrinsic characteristic of PDMS rather than an artifact of the interface. As a result, PDMS27 exhibits a more ordered interfacial structure compared to its shorter counterparts.

On the other hand, the simulated IFT results (Table 3) are in close agreement with the literature values.^18^ Specifically, the simulated IFT values for all three PDMS molecular weights with water are within 1.0 dyn/cm of each other, ranging from 39–40 dyn/cm, compared to the literature value of approximately 41 dyn/cm. This strong agreement validates both the accuracy of our simulation protocol and the chosen force field parameters, confirming that the interfacial behavior between PDMS and water is being captured well.

Overall, the data reveal a decoupling of macroscopic and microscopic properties. While chain length has little effect on interfacial tension, it plays a significant role in governing molecular-level organization and mobility. The distinct phase separation observed across all systems highlights the intrinsic immiscibility of PDMS and water. However, the greater uniformity in PDMS density with molecular weight indicates that the polymer chain length affects interfacial structuring.

#### 3.1.4 PDMS-Octane Interface

In contrast to the PDMS-water systems with sharp, well-defined interfaces, the PDMS-octane interfaces exhibit clear evidence of miscibility between the two nonpolar phases in the density profiles (Figure 6). Table 2 summarizes the interfacial thickness values, showing that the PDMS-octane systems exhibit broader interfacial thickness, indicating that PDMS and octane molecules intermix across the interface.

**Figure 6:**
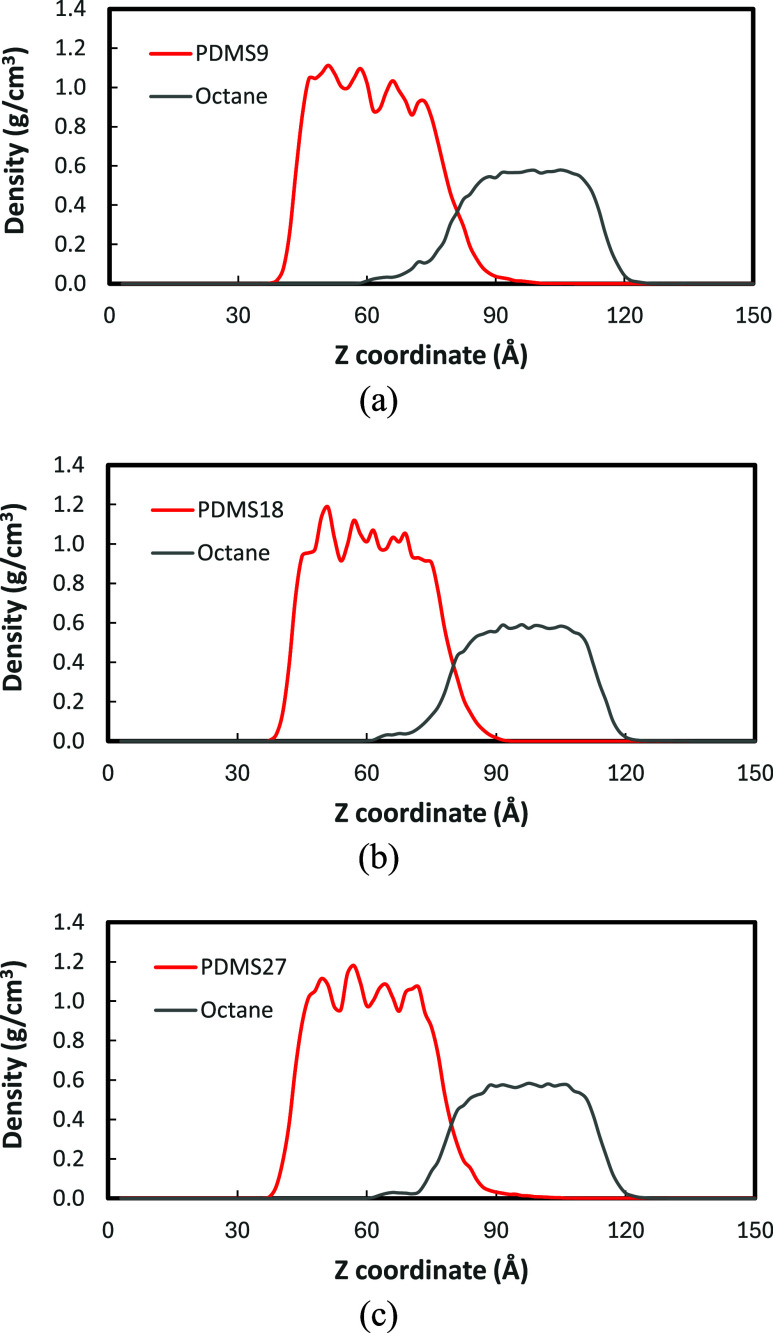
Density profiles for PDMS-octane interfaces: (a) PDMS9; (b) PDMS18; (c) PDMS27.

A molecular-weight dependency is observed at the PDMS-octane interface: the interfacial thickness decreases with increasing chain length (PDMS9:8.0 ± 2.3 Å; PDMS18:6.6 ± 4.0 Å; PDMS27:6.1 ± 3.4 Å), indicating that shorter PDMS chains penetrate and mix more readily into the hydrocarbon phase. In contrast, PDMS18 and PDMS27 exhibit a less broad interface, which is attributed to reduced conformational freedom in longer chains.

Regarding IFT, we found that PDMS-octane interface systems have significantly lower IFT values (10–12 dyn/cm) than PDMS-water systems (40 dyn/cm). This large reduction in interfacial tension confirms the miscibility between PDMS and octane, inducing the interdiffusion at the interface. Overall, while the PDMS-water interfaces form sharp, well-defined boundaries due to strong polarity contrast and high interfacial tension, the PDMS-octane interfaces exhibit gradual compositional transitions and molecular intermixing driven by chemical compatibility. The variation in interfacial thickness with molecular weight further emphasizes the interplay between chain mobility, molecular weight, and miscibility in governing the interfacial morphology of PDMS systems.

The observed independence of interfacial tension from molecular weight aligns with prior experimental findings. Although literature data covering wider ranges^18^ show a gradual convergence behavior, our results indicate that the variations in monomer count across the studied oligomeric regime (*n* = 9, 18, 27) are insufficient to drive quantitative differences in surface tension. This invariance stems from the saturation of immediate surface alignment. While the bulk molecular arrangement differs significantly, with longer chains forming layered structures and shorter chains assembling more homogeneously, the immediate interfacial conformation is conserved. Specifically, the dominant factor governing tension is the specific orientation at the boundary: parallel alignment creates a hydrophobic barrier at the water interface, while perpendicular alignment promotes permeation with octane. Because these orientations are independent of chain length at the interfacial regime, the resulting macroscopic surface tension remains nearly identical.

### 3.2 Mean-Squared Displacement Analyses

In addition to the density profiles and chain arrangements, we found that the PDMS chain mobility depends on molecular weight, which plays a key role in determining their structural organization under various conditions. Since the chain arrangement depends on thermodynamic conditions, the molecular motions are crucial in defining the equilibrium state of the interfacial system. In this section, we report the dynamic thermal motions of PDMS chains, which represent an important factor influencing the interfacial characteristics of PDMS.

To directly quantify chain motion, we performed the mean-squared displacement (MSD) analysis. Because MSD evaluates the extent of molecular displacement during MD simulations, it enables us to assess the PDMS chain mobility as a function of molecular weight. The MSD represents the average distance a particle or molecule travels over time and is calculated using the following equation:

9 $$\mathrm{MSD}\left(t\right)=\frac{1}{N}\sum_{i=1}^{N}{\left|r\left(t\right)-r\left(0\right)\right|}^{2}$$

where ***r*** (*t*) and ***r*** (0) denote the positions of particle *i* at time *t* and at the beginning of the simulation, respectively, and *N* denotes the number of particles. A higher MSD value indicates greater diffusivity.

For all MSD calculations in pure and interfacial systems, chain mobility was evaluated within a *z*-axis range of approximately 10 Å at the interface, allowing for direct comparison of interfacial environments by restricting the MSD calculation to polymer segments located near the interface.

We anticipate that MSD values will follow the trend MSD_PDMS9_ > MSD_PDMS18_ > MSD_PDMS27_. Shorter chains displace more readily due to fewer entanglements, whereas longer chains exhibit restricted motion. The analysis results are presented in Figure 7, which illustrates that the dynamic response of PDMS chains is strongly influenced by molecular weight and by interactions with surrounding solvents. For the pure PDMS phases in vacuum (Figure 7a), the MSD increases steadily with time and exhibits the expected molecular-weight dependence: shorter chains (PDMS9) display higher mobility, whereas longer chains (PDMS27) are more constrained due to increased entanglement.

**Figure 7:**
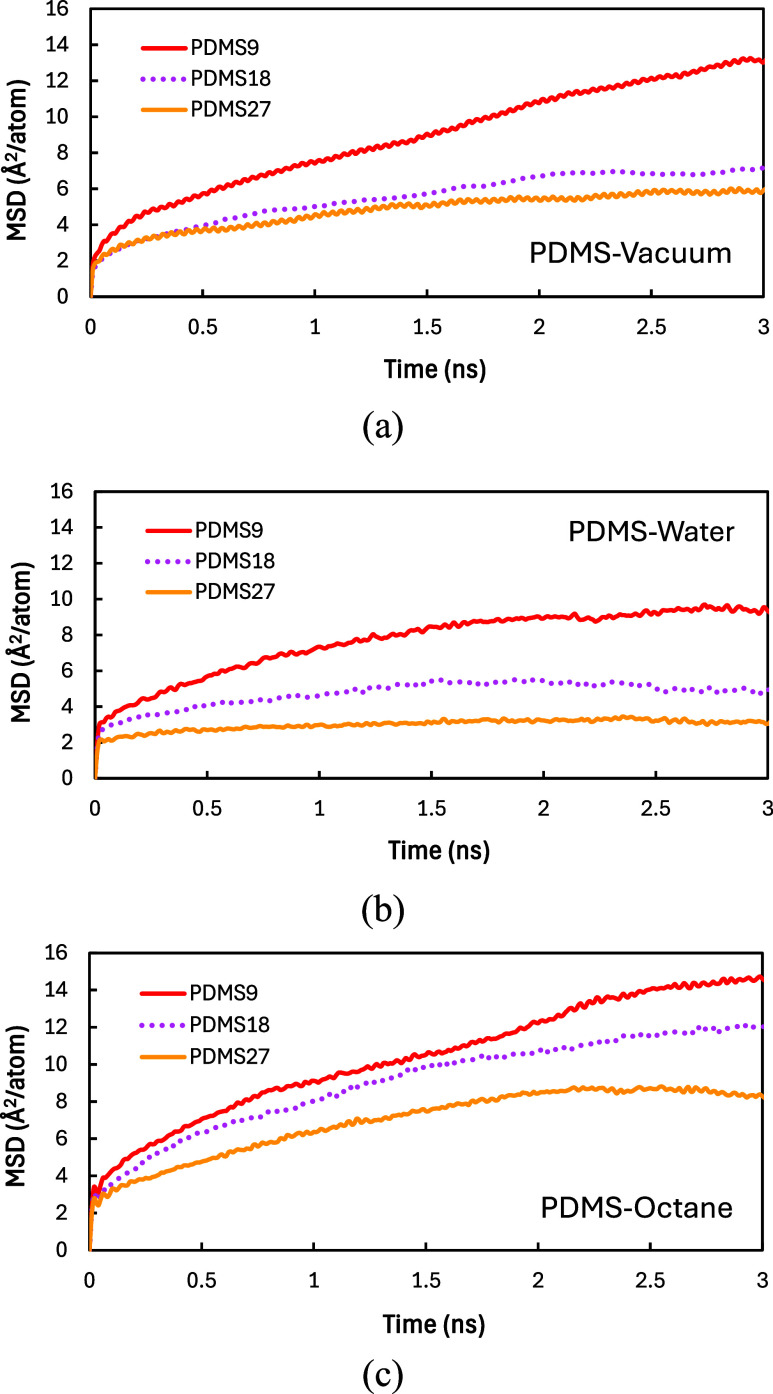
Change of mean-squared displacement for PDMS (*n* = 9, 18, and 27) as a function of time: (a) in PDMS-vacuum; (b) in PDMS-water; (c) in PDMS-octane.

In the PDMS-water interfacial system (Figure 7b), the MSD values are lower compared to the pure systems. Because PDMS is hydrophobic and immiscible with water, the PDMS-water interface is narrow, resulting in a high IFT. As a result, PDMS chains minimize contact with water molecules, reducing chain movement and displacement.

In contrast, the PDMS-octane interfacial system (Figure 7c) shows the greater overall MSD values. Since both PDMS and octane are nonpolar and chemically compatible, they exhibit high miscibility and low IFT. The PDMS chains near the interface can move freely and mix with octane molecules, resulting in larger displacements. Across all systems and molecular weights, a consistent pattern emerges: shorter chains exhibit greater mobility, whereas longer chains are more restricted.

It is important to note the consistent MSD trend among pure and interfacial systems: MSD_PDMS‑octane_>MSD_PDMS‑vacuum_>MSD_PDMS‑water_. Our MSD results highlight pronounced hydrophobic repulsion toward water and the compatibility of PDMS with octane. Miscibility with octane promotes greater molecular displacement, whereas the unfavorable PDMS-water interaction suppresses chain motion. Collectively, these results show that molecular weight, solvent compatibility, and interfacial thermodynamics together govern the dynamic phase behaviors of PDMS at interfaces.

### 3.3 Segment Orientation Analyses

To assess the equilibrium conformations of PDMS chains near the interfaces, the segment orientation profiles were averaged over the final 2.0 ns of each MD simulation. The orientation function, ⟨|cosθ|⟩, quantifies the average angle between the 5-Si segment vector of the PDMS chain and the surface normal (*z*-axis direction), where values near 1.0 indicate parallel alignment with the interface and values near 0 indicate perpendicular alignment.

#### 3.3.1 PDMS-Water Interface

In PDMS-water systems, Figure 8a shows that PDMS chains predominantly adopt horizontal orientations (perpendicular to the *z*-axis) at both the vacuum and water interfaces, consistent with the hydrophobic nature of PDMS. The horizontal alignment minimizes unfavorable contact with the polar water phase, resulting in a confined, well-ordered interfacial layer. Among the three molecular weights, PDMS9 exhibits the most significant variation in bond orientation, as reflected in fluctuations across the interfacial region, indicating a higher degree of freedom in the segment orientation. The PDMS18 and PDMS27 systems, in contrast, show more restricted orientation profiles with less variation across locations in the *z*-axis direction, indicating reduced conformational degrees of freedom and stronger orientational ordering. Despite differences in molecular weight, all three systems produce similar interfacial thicknesses (as reported in Table 2), suggesting that while chain orientations vary, the overall interfacial structural width remains relatively constant in water.

**Figure 8:**
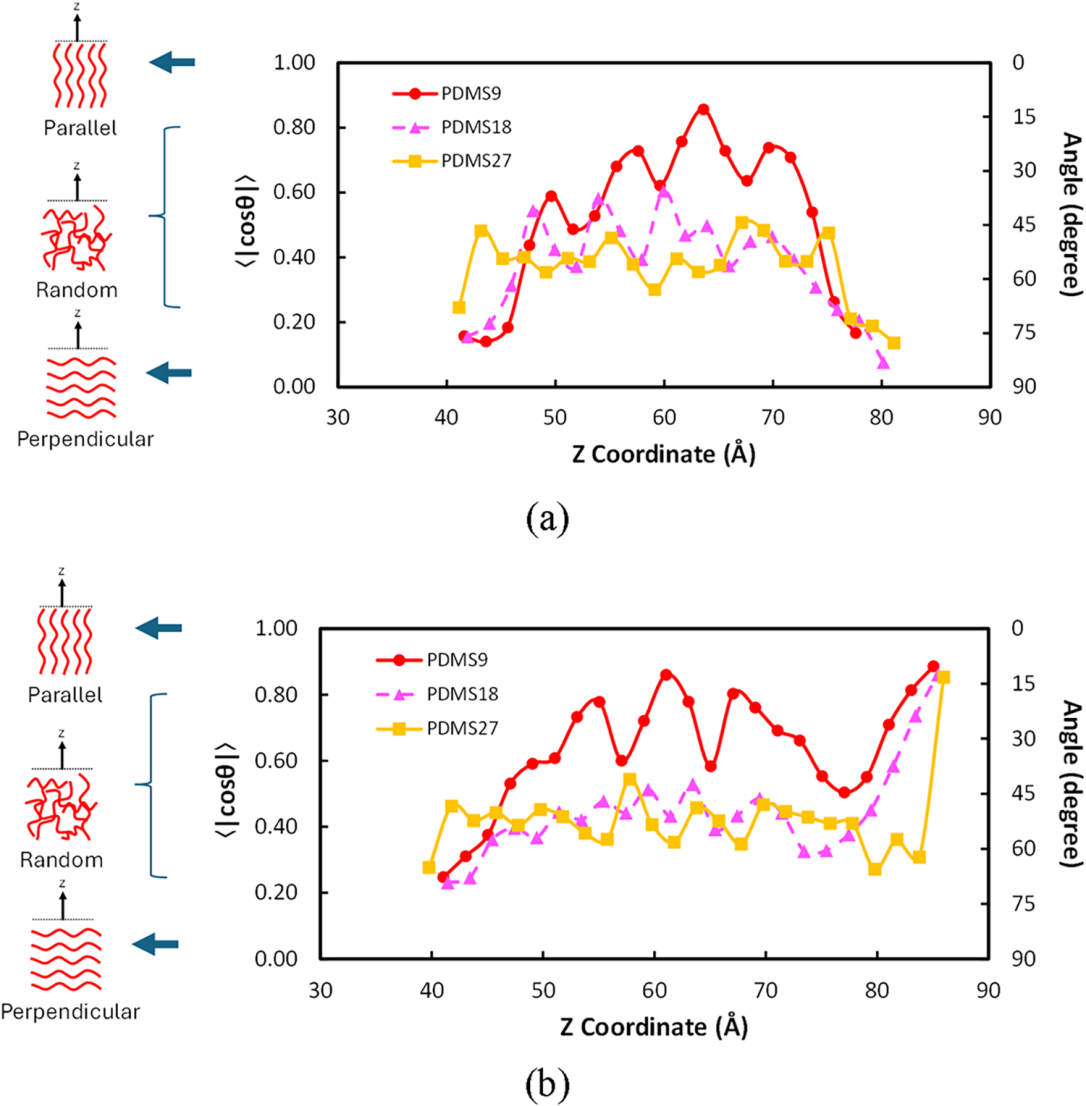
Bond orientation profile for PDMS chains: (a) PDMS-water systems; (b) PDMS-octane systems.

#### 3.3.2 PDMS-Octane Interface

In the PDMS-octane systems, a different orientation pattern emerges due to the chemical compatibility between PDMS and octane. The profiles indicate a transition from random or slightly perpendicular orientations in the bulk PDMS region to near-parallel alignment at the PDMS-octane interface. This behavior signifies greater intermixing and miscibility with the nonpolar octane phase, consistent with the lower interfacial tension observed for these systems. As observed at the PDMS-water interface, the PDMS-octane interface exhibits a similar feature: the shorter PDMS9 chains exhibit the greatest orientational variability, reflecting their ability to reorganize across the interfacial region. This enhanced variability contributes to a thicker interfacial layer, as more chains can penetrate and mix more with octane. Conversely, PDMS18 and PDMS17 show greater periodicity in their orientation profiles, with oscillations centered at 45–60°, indicating preferred chain alignment within the bulk PDMS region and more ordered configurations.

These distinct orientational preferences have direct implications for the performance of PDMS in coating and adhesive applications, as illustrated in Figure 9. A horizontal segment orientation in which chains are parallel to the interfacial plane creates a dense, protective barrier as shown in Figure 9b. This effect is more apparent in high-molecular-weight systems. In this configuration, predominant at water interfaces, the siloxane backbone shields the underlying bulk phase while maximizing the exposure of hydrophobic methyl groups. This alignment is critical for creating durable coatings, as it effectively seals the surface against water penetration.

**Figure 9:**
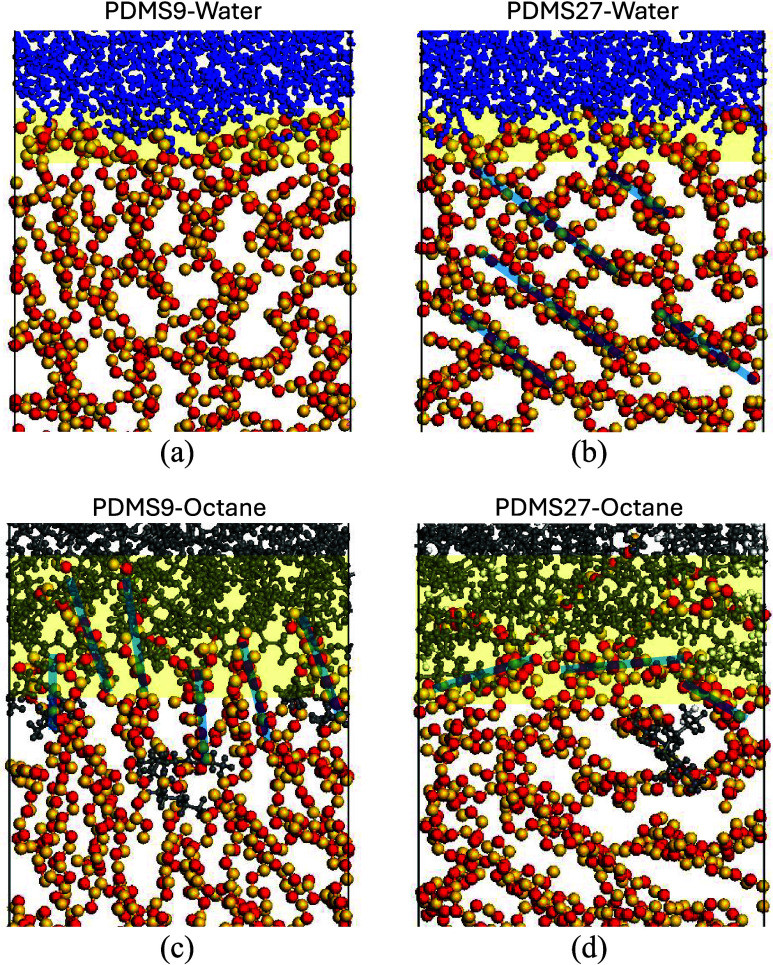
Snapshots illustrating the effect of molecular weight on interfacial structuring. (a) PDMS9-Water, (b) PDMS27-Water, (c) PDMS9-Octane, and (d) PDMS27-Octane. PDMS backbone chains are colored red and yellow (methyl groups omitted for clarity), water molecules shown in blue, and octane in dark gray. The yellow shaded regions indicate the approximate interfacial zone, while the light blue lines are superimposed to show the dominant orientation of PDMS chains.

On the other hand, a vertical chain alignment parallel to the *z*-axis, where chains adopt a “brush-like” conformation, exposes a greater interaction volume and surface area. This random, extending architecture is more present in low-molecular-weight systems. It allows for significant interpenetration with the adjacent phase, a mechanism that is essential for achieving strong adhesion to substrates and facilitating wettability by compatible solvents. Figure 9c demonstrates the adhesion capability of shorter PDMS9 chains through their octane affinity, as shown by the vertical chain configuration. This polymer phase is favoring the octane interaction, and the shorter chains readily adapt a configuration that allows octane to mix. Conversely, the PDMS27 chains have restricted motion, as shown by the “stacked” chains in Figure 9b,d. Once again, high molecular weight demonstrates the ability to uphold an interfacial separation by serving as a barrier to the interacting species, and this is a key component in protective coating design.

## 4 Conclusions

In this study, we performed comprehensive all-atom molecular dynamics simulations to elucidate how the molecular weight of PDMS influences its interfacial behavior with both polar and nonpolar liquids. Across PDMS chains of three representative molecular weights (*n* = 9, 18, and 27), the macroscopic interfacial tensions with water and octane were found to remain largely independent of molecular weight. However, the microscopic interfacial structures and dynamic responses exhibited pronounced molecular-weight-dependent characteristics, underscoring the importance of chain length in governing interfacial organization. While the local interfacial ordering observed here serves as a baseline for PDMS behavior, the distinct density fluctuations are characteristic of the oligomeric regime, which is magnified due to finite-size effects. In high-molecular-weight systems, we expect these density profiles to smooth out as chain entanglements and bulk statistical averaging dampen the density fluctuations.

Density profile analyses showed that increasing molecular weight systematically enhances structural ordering and layering within the PDMS phase, both in vacuum and at liquid interfaces. Shorter PDMS chains adopt more uniform density distributions due to greater conformational freedom, whereas longer chains exhibit pronounced oscillatory features indicative of emerging local order. At PDMS-water interfaces, all systems maintained sharp boundaries governed by the cohesive hydrogen bond network in water phase, while PDMS-octane interfaces displayed broad, diffuse interfacial regions consistent with their partial miscibility.

Dynamic analyses based on mean-squared displacement further demonstrated that chain mobility decreases monotonically with increasing molecular weight. The incompatibility between PDMS and water suppresses chain mobility near the interface, whereas the highly miscible PDMS-octane systems promote greater interfacial mixing and mobility. Orientation analyses revealed that PDMS chains adopt predominantly horizontal conformations at water interfaces to minimize energetically unfavorable interactions, while more diverse and interpenetrating orientations arise in the PDMS-octane systems.

Taken together, these findings establish that PDMS molecular weight does not significantly affect macroscopic thermodynamic quantities such as interfacial tension, but plays a crucial role in regulating interfacial structuring, segmental orientation, and chain mobility at the molecular scale. This molecular-level view provides important insights for tailoring PDMS-based coatings, adhesives, and surface-engineered materials, particularly where interfacial interactions with different classes of liquids determine performance. From a materials design perspective, our results suggest that molecular weight influences functional performance distinctly from interfacial tension. Lower molecular weights maximize the mobility required for rapid conformational response to external stimuli, such as the solvent. Higher molecular weights, however, are preferable for stability; they exploit enhanced packing density to reinforce the interface against permeation. The results also offer a refined framework for designing future PDMS systems with customized interfacial behavior and for guiding multiscale modeling efforts that connect atomistic insights to macroscopic material properties.
